# “It’s been a very, very long and emotional journey, and the impact is huge”: a reflexive thematic analysis exploring the experiences of parents of children and young people with ARFID

**DOI:** 10.1186/s40337-026-01588-9

**Published:** 2026-05-06

**Authors:** Laura Bourne, William Mandy, Rachel Bryant-Waugh

**Affiliations:** 1 Department of Clinical, Educational and Health Psychology, University College London, 1-19 Torrington Place, London, WC1E 6BT UK; 2 Maudsley Centre for Child and Adolescent Eating Disorders, South London and Maudsley NHS Foundation Trust, London, SE5 8AZ UK

**Keywords:** Avoidant restrictive food intake disorder, Children, Reflexive thematic analysis, Qualitative research

## Abstract

**Background:**

Avoidant Restrictive Food Intake Disorder (ARFID) is a relatively newly classified eating disorder which can significantly impact physical health and psychosocial function. This qualitative study aimed to explore the lived experience of ARFID caregivers in order to develop understanding of the condition and how it should be supported.

**Methods:**

Semi-structured interviews were conducted with the parents of sixteen children and young people with ARFID, who were recruited from an outpatient eating disorder service in the UK. Interview transcripts were analysed using Reflexive Thematic Analysis.

**Results:**

Qualitative analyses revealed four key themes: (1) From fussy eating to something more: The development of ARFID, (2) A fragile process: Factors that worsen and maintain ARFID (3), Developing a toolkit: Learning what helps, and (4) The weight of ARFID: The burden on the whole family. A conceptual model of ARFID development and maintenance is proposed, illustrating the relationships and interactions between the themes captured in the analysis.

**Conclusions:**

This study provides insight into the nature and course of ARFID, highlights the widespread impact on the individual and their family, and illustrates the critical role that parents play in managing this eating disorder.

**Supplementary Information:**

The online version contains supplementary material available at 10.1186/s40337-026-01588-9.

## Abstract (plain language summary)

Avoidant restrictive food intake disorder (ARFID) is an eating disorder characterised by a significant difficulty in eating an adequate range or overall amount of food, not related to weight or shape, which can have serious physical and/or psychosocial consequences. Despite a growing evidence base for ARFID, there are few studies which explore the real-world experiences of those living with this eating disorder. We interviewed the parents of 16 children and young people with ARFID, recruited from an outpatient eating disorder service in the UK. We asked a series of open questions covering a list of key topics, including the impact of ARFID on the child, main concerns for the caregiver, and treatment expectations. Following this, we analysed the transcripts and identified common themes that emerged from the interviews. Overall, the findings confirm that ARFID is a multifaceted eating disorder, which is typically driven and maintained by numerous overlapping factors. Drawing on this, we propose a model which can be tested in the future. This study explores the experience of supporting a child with ARFID, draws attention to the widespread impact that it has on the individual and those around them, and lays the foundations for future work in this field.

**Supplementary Information:**

The online version contains supplementary material available at 10.1186/s40337-026-01588-9.

## Background

Avoidant restrictive food intake disorder, or ARFID, is a diagnostic category that was first introduced to psychiatric nosology in the Diagnostic and Statistical Manual of Mental Disorders in 2013 (DSM-5; [1]) and then more recently added to the International Classification of Diseases, 11th Revision (ICD-11; [2]).

ARFID captures a cohort of individuals who eat a severely restricted diet for reasons not relating to weight, shape, or body image, which leads to a persistent failure to meet nutritional and/or caloric needs, and/or significant impairment in psychosocial functioning [1]. Research demonstrates that ARFID captures a range of different presentations which vary according to what is driving the restriction [3–5]. These include, but are not limited to, a lack of appetite or little interest in food or eating, an avoidance relating to the sensory characteristics of food, and a concern about the aversive consequences of eating [6]. This list is not exhaustive however, and the drivers contributing to the onset and perpetuation of restrictive eating behaviours frequently overlap and co-occur [7–9].

The literature indicates that caring for an individual with an eating disorder can be a significantly challenging, highly distressing and burdensome experience [10–12] and positive family involvement during the course of the illness has been shown to have a considerable impact on recovery outcomes and quality of life [13–15]. A systematic review synthesised qualitative research in relation to parents’ experiences of caring for a child with anorexia nervosa. Parents cited feelings of fear, shame, and loneliness, and guilt for contributing to the illness [16]. This has also been explored with carers of children with bulimia nervosa [17, 18]. Themes centred around the ongoing impact of caregiving, coping strategies used by the carer, and the need for guidance and support.

To date, one qualitative study has examined the experiences of adults with ARFID using semi-structured interviews [19] and two further qualitative studies provide invaluable insight into ARFID caregivers’ experiences outside of the UK, namely Australia and Sweden [20, 21]. Several studies have also qualitatively investigated the perceptions and feeding practices of those experiencing non-clinical fussy eating behaviours. Wolstenholme and colleagues [22] conducted a synthesis of ten recently published qualitative studies examining non-clinical fussy eating behaviours in children and young people with a particular focus on the perceptions and feeding practices of families experiencing such challenges.

There is, however, a lack of research relating to ARFID caregivers’ experiences in the UK. To this end, the current study aims to explore the lived experience of ARFID caregivers in the UK in order to develop understanding of the condition and how it should be supported. Specifically, this study sought to understand how parents interpret the development and maintenance of ARFID, how they support their child both practically and emotionally, and the broader impact on family life and functioning, with a view to informing treatment developments.

## Methods

### Interview participants and recruitment

We recruited a diverse sample of participants who were undergoing treatment at an ARFID clinic located within an outpatient eating disorder service for children and young people in England. Participants were deemed eligible if they were the caregiver of a child or young person (aged 2–17 years) with a diagnosis of ARFID.

All caregivers, including parents and other carers, who met the broad criteria and had already expressed a general willingness to be contacted about research studies were approached by clinicians and invited to participate. Interested participants were then provided with the necessary information to contact the research team directly.

The parents of twenty-three children and young people with ARFID were referred to the research team. All potential participants made initial contact, but seven withdrew from the study before completing the interview because of a failure to respond to follow up or return the necessary forms. In total, the parents of sixteen children took part in the interviews: fourteen mothers and two fathers (see Table 1 for demographic information). All caregivers were biological parents aged 37–58 years (*M* = 45), 69% (*n* = 11) of whom were white British, and the remainder were from a range of ethnic backgrounds, including Greek British, Irish, and Russian. Children were aged 5–17 years (*M* = 10.5) and had a current diagnosis of ARFID, with some reported additional diagnoses, namely, ADHD (*n* = 1), autism (*n* = 6), depression (*n* = 1), anxiety disorders (*n* = 2) and specific phobias (*n* = 1). Assigned sex at birth and current gender identity were consistent for all children in the sample.

Preliminary data analyses were conducted alongside data collection so the research team could consider data saturation. This was defined as the point at which additional data collection was unlikely to yield no further themes or substantially alter the findings [23]. Recruitment ceased in March 2023.

**Table 1:** Participant demographics (*N* = 16)

Sex	Female	14
	Male	2
Age (years)	35–39	4
	40–44	7
	45–49	1
	50–54	3
	55–59	1
Child sex	Female	7
	Male	9
Child current age (years)	5–8	5
	9–12	6
	13–17	5
Child age (in years) at ARFID diagnosis	2–4	1
	5–8	7
	9–12	4
	13–17	4

### Materials and procedure

The research team designed a semi-structured interview schedule lasting between 30 and 60 minutes consisting of main questions and further prompts. The interview schedule consisted of open questions covering a list of key topics pertaining to the impact of ARFID on the child, main concerns for the caregiver, and treatment expectations, for example: ‘Can you tell me about your child’s eating?’, **‘**Could you tell me about how these difficulties developed?’, ‘What do you think maintains the problem, what causes it to keep happening?’, ‘What impact does this have on your life?’ and ‘What do you think makes the problem worse?’ (see online Appendix 1 for a full interview schedule).

The question schedule was loosely observed, and prompts were used to elicit a rich account of participants’ experiences and to encourage discussion of any other topics they felt were relevant. A short demographic questionnaire was also given to participants which included questions about age, ethnicity, biological sex and relationship to child (see Online Appendix 2).

Interviews were conducted by the first author (L.B.) and took place online via video call (Microsoft Teams) at a time suitable for participants.

### Ethical considerations

This study received ethical approval by the North West - Greater Manchester South Research NHS Ethics Committee (ref. 21/NW/0072). Ethical principles were adhered to, and all parents were guaranteed anonymity, made aware of their right to withdraw, and fully briefed before and after participating in the study. Written informed consent was also obtained.

### Data analysis

All interviews were digitally audio recorded and transcribed, and personal or identifiable information was redacted. The transcripts were entered into qualitative research software, NVivo (version 14; NVivo, 2023) to aid data management and facilitate analysis.

This exploratory study employed an inductive, data-driven approach. Reflexive Thematic Analysis was used according to Braun and Clarke [24–27], which is a six-phase method used for identifying and reporting patterns of meaning within qualitative data. The reflexive aspect of the analysis recognises the active role of the researcher and acknowledges the influence of their prior assumptions or biases on the interpretation of data.

First, the lead author (L.B.) became familiar with the data by transcribing the interviews, then reading and re-reading the transcripts and noting down initial thoughts. Next, using NVivo 14, recursive line by line coding was conducted to assign descriptive labels to the data. This resulted in a list of code names, alongside descriptions and excerpts from the data. With input from W.M. and R.B.W., the codes were revised and organised into broader candidate themes with shared meaning. The candidate themes were again revised and merged until a preliminary thematic framework was established which reflected key patterns of meaning within the data. Following this, an iterative process of reviewing and refining the preliminary thematic framework was conducted with the entire research team until a consensus was reached on the final themes and subthemes.

Reflexive practice was used throughout the data collection and analysis process. Two members of the team are practicing clinical psychologists, one of whom works at the clinic where recruitment took place but had no direct contact with the parents as research participants. All team members are engaged with research. Therefore, the research team as a whole are closely positioned to the topic and were aware of their influence on the interpretation of the data. The first author (L.B.) kept a reflexive diary throughout the interview and data analysis.

The team considered the philosophical stance of the research prior to commencement of the study as this can influence the research design and interpretation of the data. Data analysis adopted a broadly critical realist framework which asserts that data informs reality but is not wholly reflective of it [28]. Instead, our understanding of the world is a construction of our measurable and observable experiences [29–32]. Participants’ accounts were considered a subjective version of the truth, shaped by their understanding of the social world, and further constructed through the researcher’s interpretive lens.

## Results

Codes were structured around four key themes with further subthemes, which pertained to the experience of living with and caring for a young person with ARFID (see Table 2): (1) From fussy eating to something more: The development of ARFID, (2) A fragile process: Factors that worsen and maintain ARFID (3), Developing a toolkit: Learning what helps and (4) The weight of ARFID: The burden on the whole family.

**Table 2:** Overview of themes and subthemes

Themes	Subthemes
1. From fussy eating to something more: The development of ARFID	a. Inherent vulnerabilities
	b. Contextual stressors that intensify restriction
	c. “A turning point”: Incidents that marked the onset
2. A fragile process: Factors that worsen and maintain ARFID	a. “I fear we made it worse”: When pressure is part of the problem
	b. External influences that destabilise progress
3. Developing a toolkit: Learning what helps	a. Practical strategies in everyday life
	b. “Creating a safe haven”
	c. “It had to be his choice”. When motivation comes from within
4. The weight of ARFID: The burden on the whole family	

### Theme 1: From fussy eating to something more: the development of ARFID

All parents reflected on the development of their child’s eating difficulties. Accounts broadly aligned with two distinct developmental trajectories: early and enduring food selectivity, and a sudden onset of symptoms linked to a traumatic or distressing trigger incident.

#### Subtheme 1a: Inherent vulnerabilities

Most parents reported that their child had particular characteristics that they believed contributed to the subsequent development of ARFID. As such, ARFID was not deemed to have emerged suddenly, but rather, developed in the context of pre-existing vulnerabilities. The most commonly cited were sensory sensitivities, which were apparent from an early age. These included sensitivities to texture, temperature, appearance, noise, and a strong disgust reaction:

*“We had further sensory issues around packaging*,* food packaging. She hated the look of it. She didn’t like me cooking. She didn’t like the sound of the kettle being boiled. Me and my husband couldn’t eat in front of her*,* so it was just this sort of real onset of everything.”* P10.

Several participants also reported longstanding difficulties with attention and sustained focus. Parents described their child as easily distracted during mealtimes, and unable to sit still and engage with their food:

*“He’s always had attention issues and struggles to sit still…we’ve never expected him to sit at the table to eat because he just can’t.”* P01.

Interoceptive awareness was also mentioned by several other parents, who described recognising and responding to hunger and satiety cues as a challenge for their child:

*“Interoception is definitely a big thing because he just doesn’t feel hunger until he’s absolutely ravenous. So*,* he’s not motivated to eat because he doesn’t feel hungry until he’s starving*,* by which time he feels so awful that he doesn’t feel like eating anyway.”* P02.

#### Subtheme 1b: Contextual stressors that intensify restriction

Alongside the inherent vulnerabilities previously mentioned, some parents noted various contextual stressors which they felt contributed to the development of ARFID. Specifically, there was a sense that the characteristics discussed in Subtheme 1a fostered fussiness around food, but that certain contextual stressors exacerbated these issues to such a degree that they became clinically significant.

Parents frequently reported age-related transitions or developmental changes, such as starting school, as worsening existing eating difficulties because of emotional over-arousal or sensory over-stimulation:

“*In school he can’t stand being around all the smells and sights of other people’s foods…he was suddenly surrounded by all the smells and sights of the hot food*,* and he hated it*,* really dreaded lunchtime.”* P03.

One parent described their child as feeling nervous around food and experiencing longstanding emetophobia. While this fear had been manageable and had not previously affected eating to a significant extent, the onset of COVID-19 fostered a fear of contamination and heightened concerns of vomiting and illness, which contributed to a marked shift towards food restriction:

*“There was a lot of heightened sensitivity*,* mask wearing*,* germs*,* hand washing*,* all of that although not evident at the time*,* is something she has since reflected on and realised it affected her fear of germs and emetophobia. She’s had a fear of vomiting since she was 6*,* but that hasn’t manifested for her in a way that was problematic on a day-to-day basis with her eating until she reached around 15. She reflected how the pandemic and the cleaning; it was just too much for her.”* P11.

For some, the act of seeking support functioned as a contextual stressor. Professional input from dietitians, health visitors or school staff resulted in increased pressure, greater anxiety and counterproductive advice which inadvertently reinforced difficulties:

*“He was just about to start school*,* and we were going to send him in with a packed lunch*,* and she [the dietitian] said don’t do that*,* don’t tell the teachers he has any kind of issues around eating*,* and he’ll soon get hungry enough that he’ll eat school dinners.”* P03.

*“At that age*,* it’s developmentally normal to be fussy so I wasn’t overly concerned and neither was the health visitor. They just gave me the universal advice to offer things and not have any pressure around eating*,* so we continued with that. But it never really improved.”* P07.

#### Subtheme 1c: “A turning point”: incidents that marked the onset

In contrast to the group above, for whom ARFID appeared to develop gradually from longstanding difficulties with food and eating, an alternative developmental trajectory was described by a smaller number of parents. Specifically, they reported its onset as sudden and event driven. In these cases, a discrete and identifiable event marked a clear shift in food acceptance. In these cases, parents emphasised a before and after contrast, recalling that prior to the incident, their child had a healthy relationship with food and exhibited very little food selectivity.

Parents identified experiences such as choking, vomiting, and episodes of illness, after which eating became associated with fear and avoidance.

*“She had a couple of incidents where she vomited in fairly dramatic circumstances - she vomited in her sleep once*,* and after an evening meal at a family party. And now we think maybe that was something that set it off*,* but you don’t really know at the time*,* it doesn’t come with a flag warning.”* P16.

*“She weaned really easily at 6 months*,* she ate anything and everything*,* whatever we had…she was a dream. And she stayed like that until she was around 6 months old. And what seemed to be the trigger was that she got really ill with a chest infection at about 18 months… when she got better*,* she would only eat soft stuff…as long as it was soft or would melt. And I don’t know if*,* in her head*,* they were things that hurt her with the tonsilitis”* P13.

### Theme 2: A fragile process: factors that worsen and maintain ARFID

Parents reflected on the delicate nature of ARFID, and the challenge of managing factors that could destabilise progress. This theme encompasses both interactional responses between the parent and child and external stressors that unintentionally reinforce restriction.

#### Subtheme 2a: “I fear we made it worse”: when pressure is part of the problem

There was universal agreement that the application of pressure was counterproductive. This included coercive tactics to encourage the child to try new things, pressure to eat larger quantities, and more generally, heightened scrutiny during mealtimes:

*“We went through a phase of having super stressful mealtimes*,* you know the pressure of getting her to have another bite*,* and she said she used to feel the dread before a meal.”* P09.

*“If I try and force it*,* it goes completely the other way*,* and then he won’t have anything.”* P05.

Relatedly, conflict or tension at mealtimes, and in particular, disagreements with another parent or carer about how best to tackle the issue was a source of tension, resulting in reduced mealtime engagement or a complete refusal to eat:

*“We argue about him using his iPad at the dinner table. I see it as a necessity*,* but his dad will kick off if he’s there. And then we get complete shutdown. It’s traumatic.”* P01.

#### Subtheme 2b: External influences that destabilise progress

In addition to interactional factors within the family, parents also noted external influences that destabilised progress. Periods of illness were frequently cited as a source of concern, as they would often result in increased dietary restriction. For some, this was related to a loss of appetite accompanying the illness and for others, this prompted negative associations with food eaten around the illness onset. Elimination of foods following illness had disproportionate consequences, diminishing an already limited repertoire of “safe foods” and highlighting the precarious nature of recovery in ARFID:

*“Illness is the big thing - if he becomes ill while he’s eating a certain food*,* that’s it*,* it’s gone forever. He will associate that with being ill. That food made me sick so now I can’t trust it.”* P12.

Disruptions to established routines, for example, school holidays, were frequently reported to precipitate a period of regression:

*“Any setbacks*,* changes to routine*,* summer holidays is a big struggle not having school…as time goes on*,* he will just shut down*,* and I’ve had it where he won’t eat for weeks. It causes him trauma and we lose the eating.”* P04.

Finally, parents mentioned more nuanced maintaining factors, which were tied to the child’s specific fears and sensitivities. For one child with an intense fear of vomiting, particular words or phrases could trigger a setback:

*“Anyone who mentioned feeling sick or ill*,* people use it quite interchangeably of course*,* they might mean they’ve got a cold*,* but that was incredibly alarming for her*,* she would go into panic*,* she wouldn’t eat.”* P11.

### Theme 3: Developing a toolkit: learning what helps

Theme 3 captures parents’ perspectives on things they have found to be helpful in supporting improvements in their child’s eating and managing stress around mealtimes.

#### Subtheme 3a: Practical strategies in everyday life

Almost all parents discussed practical adaptations they found useful in helping to accommodate their child’s eating difficulties. The use of screens and similar distractions was frequently mentioned as a tool to reduce over-stimulation or over-arousal at mealtimes:

*“Say we want to go to a restaurant*,* we just give him his tablet or a phone to play with to distract him*,* and he can quite happily sit in a restaurant…if we want to go out as a family*,* you can distract him from the panic*,* because he will get overwhelmed and upset.”* P03.

Structure, routine and preparation were vital in fostering a sense of control for the parents and a sense of safety for the child. Packed lunches were frequently cited as an essential tool which functioned as a practical strategy to facilitate participation in social events that would otherwise have been avoided because of concerns around food:

*“School is packed lunches; he has a very specific set of accepted foods for that. At home*,* everything revolves around accepted foods.”* P14.

Relatedly, there was a sense that offering mostly accepted and familiar foods was key to ensuring steady progress and maintaining trust:

*“As long as we’re able to give him the things he likes*,* he will eat to sustain growth and have enough energy*,* there’s just not a lot of variation. The dietitian said it’s good enough in terms of maintaining growth*,* and then he needs a multivitamin alongside. So*,* I feel more confident.”* P07.

#### Subtheme 3b: “Creating a safe haven”

Many parents emphasised the importance of the home environment and in particular, ensuring a calm and emotionally unpressurised “safe zone”. Central to this was removing pressure at meal and snack times, replacing coercion with gentle encouragement, and fostering a sense of autonomy:

*“Just taking the pressure off anything at home*,* so keeping home as the real safe zone*,* you know*,* giving her safe foods*,* not trying to overwhelm her with things.”* P10.

For one parent whose child had developed a fear of food contamination, nurturing trust through honesty and transparency was key to encouraging progress:

*“We spoke about how she had to trust us*,* and watch us cook her food*,* to reduce the fear of germs. She’d wanted it cooked in a certain way; make sure it was clean. So*,* trust is a big thing*,* she needs to trust us that we’re giving her good food that won’t make her unwell.”* P11.

#### Subtheme 3c: “It had to be his choice”. When motivation comes from within

Several parents observed a shift in their child’s intrinsic motivation which appeared to mark a turning point in progress. For those entering later childhood or adolescence, this was often linked to increasing social awareness which motivated a desire to participate in peer activities, share meals socially and to integrate at school. Unlike external direction or parental pressure, this internally mediated drive proved to be particularly effective in facilitating change:

*“She has got a lot better with her friends…in the last couple of months*,* they’ve started doing a Friday night sleepover and interestingly enough*,* sometimes they cook…she’s started doing a bit of cooking and actually she seems to take the lead on that which is really interesting. I think it’s about being in control*,* even if it’s trying something new*,* and with them she tries more things…it was a miracle.”* P08.

One participant reflected on her son’s new romantic relationship, which brought about positive pressure to try new foods and eat out at restaurants:

*“She’s a 17-year-old girl who likes to do what she likes to do*,* and she puts him under pressure to go out and eat. Which she has done. The motivation is there because he’s obsessed with this girl*,* and she sits there and eats what she likes*,* and he sits and has chips. And it doesn’t seem to bother either of them. In a way it’s been quite a positive thing.”* P02.

### Theme 4: The weight of ARFID: the burden on the whole family

The final theme captures the perceived impact of ARFID, which was a central topic of discussion for all parents. Conversations centred around the immediate consequences of ARFID, as well as concerns for the future. Discussions related to personal impact on the parents themselves, as well as broader repercussions felt by the family.

On a practical level, parents described the burden of pressure they felt in ensuring that accepted foods were available. This involved careful planning and preparation, particularly for social events, days out, or holidays and that food was prepared in advance for days out or holidays. The responsibility of food management and the experience of preparing food and pre-empting difficulties was described as “emotionally exhausting” and time consuming.

*“When we book a holiday*,* I have to make sure I book a catering apartment so we can cook for her. Every day*,* every outing*,* every holiday you’ve got to think about how I can make sure she’s got the things she needs. You know*,* the terror when the thing she’s eating is not stocked in the supermarket.”* P10.

There was also some mention of ARFID impacting family life indirectly, for example, by hindering opportunities to spend time together:

*“On a Saturday we’d love to go out for breakfast or lunch*,* it’s just a nice social family thing to do. But that’s been taken away.”* P10.

Beyond the practical impact, the emotional toll of supporting a child with ARFID was well cited. Parents expressed a range of emotions from guilt and frustration to worry and isolation:

*“It’s just really stressful to watch your child not willing to eat anything.”* P05.

Finally, the indirect impact on siblings was touched upon by several parents. One parent described the juggle of managing the needs of their son with ARFID, whilst respecting and acknowledging his sister’s preferences:

*“He gets his accommodations*,* so it gets tricky when his sister says she doesn’t like things. I need to make sure I’m respecting her preferences because to her*,* it looks like he gets to have what he wants. So*,* it’s just navigating that and not narrowing her range of foods*,* because she sees that he can refuse things easily*,* so why can’t she.”* P07.

Another parent reflected on her son’s internalised concern for his sister, further illustrating the wider impact on the family:

*“He worries himself sick over it. He wrote a letter to Santa that I found saying that he was really worried about his sister*,* and could Santa fix it?”* P13.

#### Model of ARFID development

**Fig. 1:**
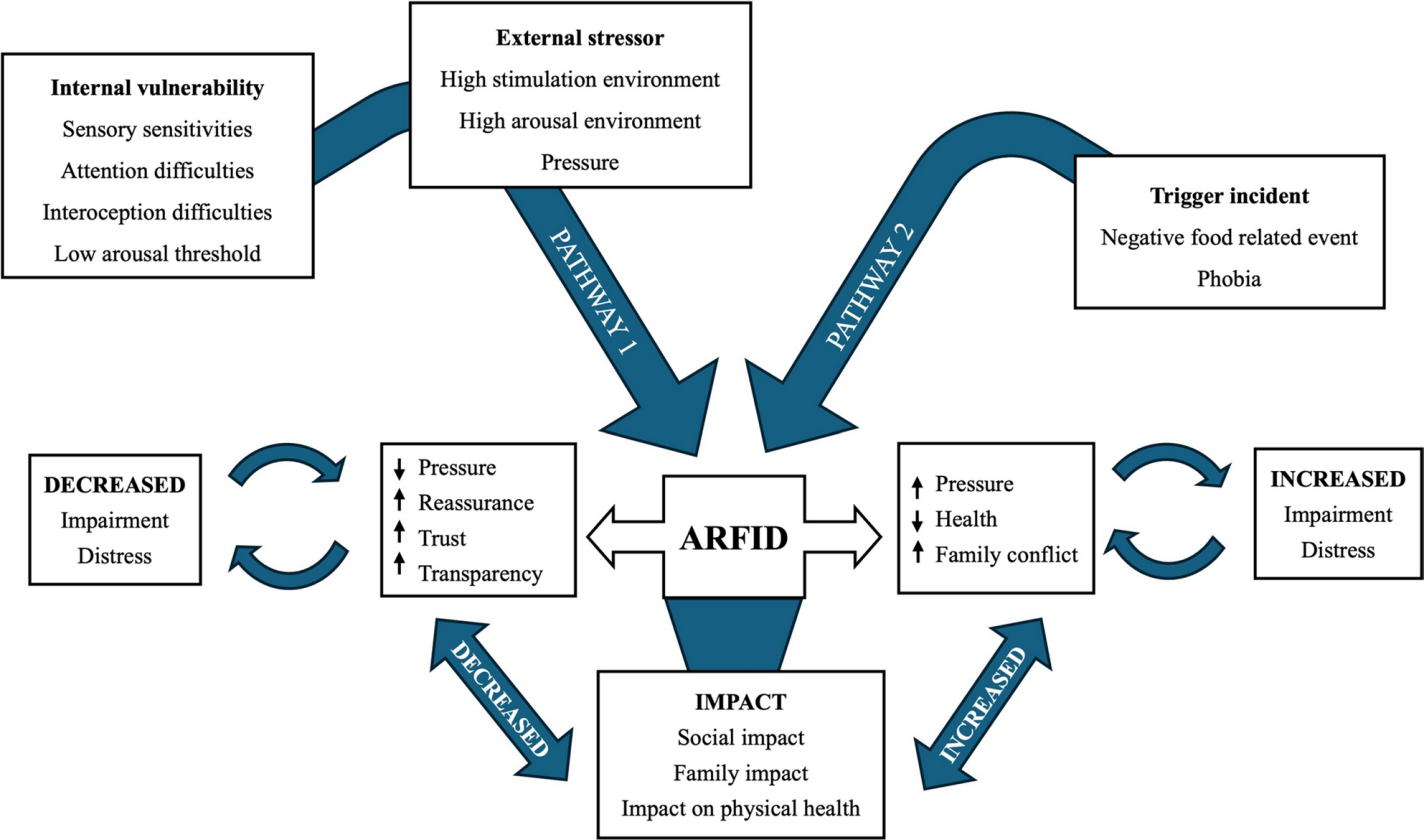
Proposed model of ARFID development and maintenance

Drawing on this data, we present a conceptual model of ARFID development and maintenance (Fig. 1). We consider this to be a set of hypotheses, derived from our qualitative analysis, for future testing.

The model illustrates two potential developmental trajectories of ARFID. The first, a gradual escalation that arises from the interaction between inherent vulnerabilities and contextual stressors. For example, sensory sensitivities may give rise to a limited diet as a result of preferences based on the sensory qualities of foods. While such characteristics alone are likely manageable and may simply result in food fussiness or idiosyncratic preferences, we suggest that a contextual stressor or set of stressors, such as a high stimulation (sensory) or high arousal (emotional or attention) environment, could intensify or exacerbate such behaviours and further reduce dietary intake, resulting in clinically significant restrictive eating concerns.

The second pathway identified from the data is via a discrete event, trauma, or internal overarousal which prompts a sudden or acute onset of symptoms. While we recognise that the above-mentioned predisposing characteristics thought to foster food restriction may be present in anyone presenting with ARFID whatever the pathway of development, we propose that the primary drivers underlying the two pathways, along with treatment approaches and outcomes, are inherently different.

Central to the model are dynamic feedback loops which can perpetuate food restriction or facilitate improvement. For example, parents reported that by reducing mealtime pressure and promoting trust, transparency, and reassurance, they noticed a decrease in their child’s distress and impairment. Consequently, caregivers felt yet more trust in the process, which further reduced pressure around mealtimes, and boosted the level of reassurance and transparency they could offer to their child. As impairment and distress reduced, so too did the impact of ARFID. In contrast, families discussed positive feedback loops, for example, where an increase in pressure on the child, family conflict, particularly during mealtimes, and instances of illness increased impairment and distress. As a result of this increase, parents responded with increased pressure, and conflict worsened, thus increasing the impact of ARFID.

## Discussion

This qualitative study aimed to explore the lived experience of ARFID caregivers in the UK in order to develop understanding of the condition and how it should be supported. Four key themes and further subthemes were identified pertaining to the development of ARFID, sources of escalation, management of symptoms and impact on the parent and family life. Importantly, while general themes were found to run through the data, the experiences of those with ARFID were seen as distinct and variable, in line with the heterogenous phenotype of the condition itself [4, 33].

The findings provide a nuanced insight into the lived realities of supporting a child with ARFID. Parents frequently described feelings of guilt regarding the nutritional quality of accepted foods, which was compounded by judgement from family members, other parents and professionals. Well-intentioned but unhelpful guidance was found to function as a reinforcer of avoidance, whereas for some, social pressure and peer influence were cited as positive drivers of improvement, particularly when experienced as internally motivating rather than coercive. This is in contrast to existing literature, which frequently highlights the potentially negative impact of peer pressure on eating behaviours [34]. These findings emphasise the need for greater professional and public awareness of ARFID and suggest that social motivation may play a valuable role in treatment.

Participant perspectives indicated that ARFID is highly impactful. Parents described their own emotional distress, the bearing on familial relationships, and the practical implications of supporting someone with ARFID. This is in line with research which evidences the significant challenge and burden of caring for an individual with an eating disorder [10–12]. Importantly, the findings from this study suggest that the family can have a significant impact on ARFID symptomology, for example, increased pressure at mealtimes can further increase distress. This has been further evidenced by Prasetyo and colleagues [35], who found that the caregivers’ behaviours, confidence in their abilities, and the home environment played a key role in supporting the child through ARFID treatment. This has important implications for treatment and suggests that an individualised approach, which is tailored to the needs of the child but also involves the family through parent training, support and practical advice may be beneficial.

Notably, treatment approaches that parents found helpful appeared to contrast with those used to promote recovery in anorexia nervosa. Anorexia interventions often involve a degree of mealtime pressure to increase food intake and dietary variety (i.e [36]), , and encourage cognitive flexibility and adaptability to change [37, 38] In contrast, participants in the current study reported that a reduction in mealtime pressure was beneficial to encouraging eating, and that routine, consistency and predictability were more effective for encouraging adequate food intake in ARFID. Thus, while ARFID and anorexia nervosa may appear symptomatically similar, particularly in those who exhibit significant weight loss [39], the findings from this study support the view that the underlying drivers are fundamentally different and therefore, the two require different treatment approaches. This also supports the literature which discusses the impact of a misdiagnosis of anorexia nervosa for neurodivergent eating disorder patients who may more appropriately receive a diagnosis of ARFID [40, 41].

We also propose a conceptual model which draws on our findings and illustrates the relationships and interactions between the themes captured in this study (Fig. 1). The model demonstrates the heterogeneous nature of ARFID development and maintenance, and highlights the value of appropriate family involvement, parental self-efficacy, and consideration of the emotional and sensory environment. As part of this model, we identified two broad pathways of ARFID development. It is important to note, however, that there was unique variation within these pathways, with perceived contributing factors presenting in different severities and combinations.

The current model aligns somewhat with the three-dimensional model of ARFID described by Thomas and colleagues [8], further supporting the conceptualisation of ARFID as a heterogeneous and dimensional condition. Parents’ accounts reflected considerable variability in presentation, with restrictive eating often shaped by an interplay of multiple underlying drivers. Notably, the current model tentatively proposes an alternative developmental trajectory, described by Pathway 2, which may represent a comparatively distinct presentation. This pathway appears to capture a subset of individuals with ARFID with no prior or longstanding difficulties with food or eating, who experience a sudden and acute onset of symptoms following an identifiable trigger event.

### Strengths and limitations

To our knowledge, this is the first qualitative study to explore the lived experience of those living with and caring for a child or young person with ARFID in the UK. Therefore, it addresses a critical gap in the field [42].

There are, however, several limitations to the present study. First, participants were recruited from a single ARFID clinic located within an outpatient eating disorder service for children and young people in England. Given that all participants were actively receiving clinical support, there is a question as to whether the experiences of families at different points in the support seeking or recovery journey may differ. It is also important to consider whether engagement with ARFID treatment contextualised the experiences of those who took part. For example, psychological formulations given to participants during the course of their treatment could have structured their experiences.

There is also a need to reflexively engage with the process and to consider the position of the research team, all of whom are familiar with ARFID literature, and fully engaged with practice, research, or both. Consistent with Braun and Clarke’s Reflexive Thematic Analysis framework [24–27], significant efforts were made to acknowledge pre-existing interpretations and assumptions via regular reflexive discussion and journaling. Nonetheless, consideration should be given to the positionality of the research team, and the extent to which this may have shaped the conceptualisations and analytic narrative that was presented. Conversely, none of the research team had lived experience of ARFID or caregiving in this context. This may have been useful for adding further nuance to the interpretation of the data.

### Implications and recommendations

This research supports the current view of ARFID as a heterogeneous disorder underpinned by numerous and varying interacting elements. Continued qualitative exploration is needed to further elucidate these mechanisms, and to ensure that patient perspectives are meaningfully integrated into evidence-based practice.

The current findings are based on parental perceptions so do not fully capture the experiences of the young people themselves. As such, the results are likely to be context dependent and future research is warranted with ARFID patients directly, including those who are yet to receive a diagnosis or access to treatment, and those in alternative clinical settings, such as higher-level residential care. It would also be valuable to explore the views of patients across the lifespan. Insight into the perspectives of adults with ARFID could make an important contribution to understanding, by examining longer term social and occupational outcomes, as well as the broader health implications.ettin

Finally, this study highlights the critical role that caregivers play in managing ARFID, and the widespread impact it can have on family relationships and the home environment. As such, the findings suggest that parent training is key in targeting the beliefs and emotions around caring for someone with ARFID and equipping families with the necessary skills, knowledge and confidence to implement interventions at home. Relatedly, this adds weight to the use of gentle encouragement, reduced pressure, and the promotion of flexible treatment suited to the needs of the individual and their family.

## Supplementary Information

Below is the link to the electronic supplementary material.

Supplementary Material 1.

Supplementary Material 2.

## Data Availability

The qualitative dataset generated and analysed during the current study is not publicly available because it contains sensitive, personal information. Participants did not provide consent for this data to be shared with third parties.

## Abbreviations

ARFID: Avoidant/restrictive food intake disorder
ADHD: Attention deficit hyperactivity disorder
DSM: 5-Diagnostic and Statistical Manual of Mental Disorders, Fifth Edition
