# Supplementary material for: “It’s been a very, very long and emotional journey, and the impact is huge”: a reflexive thematic analysis exploring the experiences of parents of children and young people with ARFID
Source: J Eat Disord. 2026 May 6;14:145. doi: 10.1186/s40337-026-01588-9 (PMC13321554; doi:10.1186/s40337-026-01588-9)
Supplement: Supplementary file 1 — Supplementary Material 1. [file 40337_2026_1588_MOESM1_ESM.docx]

**Appendix 1**

**Interview schedule**

During this interview, I will be asking you questions about your child’s eating difficulties. These include questions about the nature of the issue, what you think started it and what keeps it going. I will also be interested in your hopes for treatment and recovery, any concerns that you have and the impact that this has on you and your family. At the end, I will check with you to see if there are any other topics that you think we might have missed and would like to discuss. It’s important to know that you do not have to answer any questions that you do not feel comfortable with and can stop the interview at any point. There are no right or wrong answers, only your answers. This interview will last around 45 minutes.

**TOPIC**

**Main questions**

- Follow up questions/prompts

**BACKGROUND**

**Could you tell me about your child’s eating? (Description of the problem)**

- Do you think your child consumes an adequate amount of food?
- Do you think your child consumes an adequate variety of food?
- Is your child dependent on any supplementation (i.e., oral nutritional/enteral)?

**Why do you think your child’s eating is like this? (Understanding of the problem)**

- Could you tell me about how these difficulties developed? (What do you think triggered the problem?)
- What do you think maintains the problem, what causes it to keep happening?
- Since you have been seeking treatment, has your understanding of your child’s eating difficulty changed? (Do you now think there may be different reasons as to why the problem began and continues to be an issue?)

**Prior to your current treatment, had you sought help with these difficulties?**

- What did that involve?
- Did it have any effect on the issue, either positive or negative?
- Did it have an impact on you as a parent/carer? (increased burden, worry, for example)

**What do you think makes the problem worse?**

- Why?

**What do you think makes the problem better?**

- Why?
- (if nothing) do you think there is anything that can be done (that isn’t already being done) which may improve the problem?

**CONCERNS**

**What are your main concerns about your child’s eating difficulties?**

- Are you concerned about your child’s physical development?
- Are you concerned about your child’s nutritional intake?
- Are you concerned about your child’s personal life or social relationships as a result of this issue?
- Does this issue raise concerns about family life?

**IMPACT**

**What impact does this have on your child’s life?**

- Is there anything that it stops him/her doing?

**What impact does this have on your life?**

- Does it affect other family members or those close to you?

**[If it doesn’t or has very little impact] are there any ways that you or your family have adjusted things to accommodate your child’s eating difficulty?**

- Do you do anything differently to make things easier or possible for your child? i.e., ringing in advance of a playdate to ensure that suitable food is on offer, obeying rules about what, when and where specific foods are eaten.

**COVID-19**

**Has the Covid-19 pandemic affected your child’s eating difficulties?**

- How?
- Has it improved/worsened the situation?
- Have you found it difficult to buy/get access to the foods that your child is willing to eat?

**Have your usual support services met your/your child’s needs during Covid-19?**

- How have things changed (i.e., online clinics)

**TREATMENT AND RECOVERY**

**Suppose you had a magic wand and by waving that wand, you could make things better, what would you notice that’s different?**

- What do you hope to achieve from treatment?
- What does recovery look like for you?

**FINAL REMARKS**

**Is there anything else you’d like to mention that we have not had a chance to discuss?**

**Do you have any questions?**
