# Supplementary material for: “It’s been a very, very long and emotional journey, and the impact is huge”: a reflexive thematic analysis exploring the experiences of parents of children and young people with ARFID
Source: J Eat Disord. 2026 May 6;14:145. doi: 10.1186/s40337-026-01588-9 (PMC13321554; doi:10.1186/s40337-026-01588-9)
Supplement: Supplementary file 2 — Supplementary Material 2. [file 40337_2026_1588_MOESM2_ESM.docx]

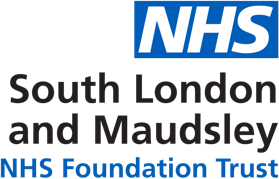

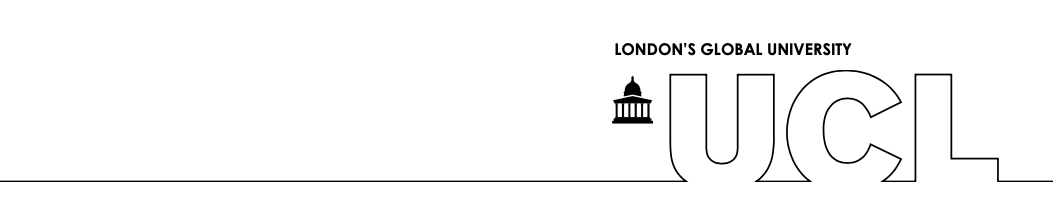


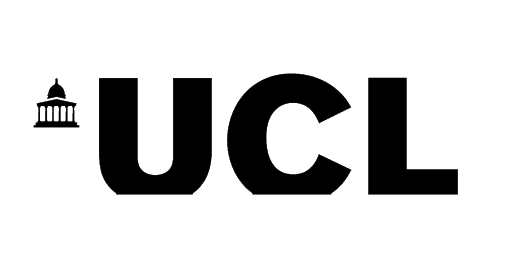


EXPLORING THE CAUSES AND CONSEQUENCES OF AVOIDANT/RESTRICTIVE FOOD INTAKE DISORDER

**DEMOGRAPHIC QUESTIONNAIRE - PARENTS AND CARERS**

**Please provide the following information about you and your child:**

**Your current age**

_________________________________________________________

**Your biological sex (and identified gender if different)**

__________________________________________________________

**Your ethnicity**

__________________________________________________________

**Your relationship to the child taking part in the study**

__________________________________________________________

**Your child’s current age**

__________________________________________________________

cont.

**Your child’s biological sex at birth (and identified gender if different)**

__________________________________________________________

**Your child’s ethnicity**

__________________________________________________________

**Year or age when your child received a diagnosis of ARFID**

__________________________________________________________

**Does your child have any other reported medical and/or mental health conditions? If yes, please list below.**
